# Influencing factors of obesity in community patients with deficit schizophrenia: a cross-sectional study

**DOI:** 10.1186/s40001-022-00706-y

**Published:** 2022-06-11

**Authors:** Na Yong, Jiyang Pan, Xuehua Li, Ling Yu, Xin Hou

**Affiliations:** 1 Mental Health Center, The Affiliated Hospital of North Sichuan Medical College, Sichuan 637000 Nanchong, China; 2 Department of Psychiatry, The First Affiliated Hospital of Jinan University, No. 613 Huangpu Avenue West, Tianhe District, 510632 Guangzhou, China

**Keywords:** Obesity, Community, Schizophrenia, Management

## Abstract

**Background:**

Obesity is very common in patients with schizophrenia. We aimed to evaluate the influencing factors of obesity in community patients with deficit schizophrenia, to provide implication for schizophrenia management in community.

**Methods:**

We selected patients with deficit schizophrenia who lived in 10 communities in our city from March 1 to June 30, 2021. The characteristics of included schizophrenia patients were evaluated and analyzed. Pearson correlation analysis was conducted to evaluate the obesity and related characteristics. Logistic regression analyses were conducted to assess the risk factors of obesity in patients with schizophrenia.

**Results:**

A total of 284 patients with schizophrenia were included, the incidence of obesity in patients with schizophrenia was 56.70%. gender (*r *= 0.619), waist circumference (*r* = 0.644), BMI (*r* = 0.891), diabetes (*r* = 0.698), FG (*r* = 0.582), triglyceride (*r* = 0.618), HDL-C (*r* = −0.644), LDL-C (*r* = 0.583), apolipoprotein B (*r* = 0.595), and PANSS score (*r* = 0.813) were all correlated with the obesity in patients with schizophrenia (all *p* < 0.05). Logistic regression analysis indicated that female (OR 2.129, 95% CI 1.615–3.022), waist circumference ≥ 90 cm (OR 3.814, 95% CI 2.778 ~ 4.312), diabetes (OR 2.856, 95% CI 1.905 ~ 3.448), FG ≥ 88 mg/dL (OR 1.551, 95% CI 1.284 ~ 2.183), triglyceride ≥ 160 mg/dL (OR 1.804, 95% CI 1.236–2.845), HDL-C ≤ 0.8 mmol/L (OR 2.032, 95% CI 1.614–3.079), LDL-C ≥ 2.0 mmol/L (OR 1.926, 95% CI 1.442–2.041) and apolipoprotein B ≥ 0.70 g/L (OR 2.119, 95% CI 1.658–2.873) were the risk factors of obesity in patients with schizophrenia (all *p* < 0.05).

**Conclusions:**

The obesity rate of patients with deficit schizophrenia in the community is high, and there are many associated risk factors. Early intervention targeted on those risk factors are warranted to reduce the obesity in schizophrenia patients.

## Background

Obesity has become a worldwide topic and a public health problem worldwide. In many countries, more than half of adults have gained weight or are obese [1]. With the development of the world economy and changes in residents' lifestyles, the incidence of obesity is on the rise worldwide [2]. Obesity is closely associated with cardiovascular disease, metabolic syndrome, and death [3, 4]. Obesity in patients with schizophrenia is very common, which increases related all-cause mortality and can reduce life expectancy by about 20 years [5]. In recent years, the relationship between schizophrenia and obesity has received extensive attention. Kassem et al. [6, 7] have found that the incidence of obesity among long-term hospitalized patients with schizophrenia is as high as 73.3%. Therefore, reducing obesity in schizophrenia patients is of great significance to the prognosis of patients [8].

At present, the research on schizophrenia is mostly limited to clinical hospitals. Deficit schizophrenia has primary and lasting negative symptoms as the main clinical feature [9, 10]. It is considered to be a highly homogeneous subtype of schizophrenia with long-term stability and high recognition rate [11]. Therefore, we aimed to investigate the obesity status of patients with deficit schizophrenia in the community and its related sociodemographic and clinical factors, to provide reliable evidence for the treatment of patients with deficit schizophrenia.

## Methods

### Ethics

In this study, all methods were performed in accordance with the Strengthening the Reporting of Observational Studies in Epidemiology (STROBE) Statement [12]. The present study had been checked and verified by the ethical committee of the First Affiliated Hospital of Jinan University (approval number: MED19008), and written informed consents had been obtained from the patients or the guardians of included patients.

### Patients

This study selected patients with deficit schizophrenia who lived in 10 communities in our city from March 1 to June 30, 2021. There are 9 administrative regions in our city, 5 administrative districts are randomly selected and we randomly selected 5 administrative regions two communities from each region to conduct a survey. The selection criteria for patients were as following with reference to previous studies [13, 14]: patients were ≥ 18 years old; the patients were assessed by the Chinese version of the Diagnostic Scale for deficit Schizophrenia and meet the diagnostic criteria for deficit schizophrenia. The main assessment items of SOS were negative symptoms (emotional limitation, speech poverty, narrow emotional range, decreased interest, decreased purpose, decreased social drive); the patients lived in the community for more than 1 year, the patient or their family members knew and agreed to participate in this study. We excluded breastfeeding and pregnant women, schizophrenia patients with mental retardation, epilepsy, brain tumors, drug abuse. According to the guidelines issued by the Chinese Obesity Working Group, this study defined obesity with a body mass index (BMI) ≥ 28 kg/m^2^ [15].

### Data collection

Two authors used self-compiled formula to collect the patient's gender, age, sociodemographic characteristics, smoking behavior, etc., and collected the patient's course of disease, age of onset, alcohol drinking, smoking, hypertension and diabetes from the patient's medical card record. We measured the weight and height of the enrolled patients, and calculated the patient's BMI. We collected patients’ morning fasting venous blood to detect the fasting glucose (FG), triglyceride, high-density lipoprotein cholesterol (HDL-C), low-density lipoprotein cholesterol (LDL-C), total cholesterol, apolipoprotein A1, apolipoprotein B, C-reactive protein, uric acid, creatinine, urea nitrogen, total protein, albumin and globulin. Additionally, we used the Positive and Negative Symptom Scale (PANSS) to assess patients' psychiatric symptoms, which had good reliability and validity in the Chinese population [16].

### Statistical methods

We used SPSS 22.0 statistical software to perform data analysis. Measurement data were expressed as mean ± standard deviation, count data were described as percentages, and the comparison of rates was performed by Chi-square test; the statistical comparison of continuous variables was performed by *t* test [17]. Pearson correlation analysis was conducted to evaluate the obesity and related characteristics. Logistic regression analyses were conducted to assess the risk factors of obesity in patients with schizophrenia. In this study, *P* < 0.05 was considered as the difference between the groups was statistically significant.

## Results

### The characteristics of included patients

A total of 284 patients with schizophrenia were included, of whom 161 patients were diagnosed with obesity. The incidence of obesity in patients with schizophrenia was 56.70%. As indicated in Table 1, there were significant differences in the gender, waist circumference, BMI, diabetes, FG, triglyceride, HDL-C, LDL-C, total cholesterol, apolipoprotein B and PANSS score between obesity and no obesity patients (all *p* < 0.05). There were no significant differences in the age, alcohol drinking, smoking, hypertension, drug treatment, course of disease, apolipoprotein A1, C-reactive protein, uric acid, creatinine, urea nitrogen, total protein, albumin and globulin (all *p* > 0.05).

**Table 1:** The characteristics of included patients

Variables	Obesity group (*n* = 161)	No obesity group (*n* = 123)	*t*/*χ*^2^	*p*
Age (years)	51.43 ± 7.91	50.77 ± 8.07	4.328	0.084
Male/female	64/97	78/45	1.216	0.004
Waist circumference (cm)	101.81 ± 26.16	81.94 ± 20.33	9.371	0.001
BMI (kg/m^2^)	29.95 ± 1.09	22.61 ± 2.15	2.144	0.003
Alcohol drinking	54 (33.54%)	47 (38.21%)	1.302	0.102
Smoking	48 (29.81%)	33 (26.83%)	2.925	0.114
Hypertension	46 (28.57%)	36 (29.27%)	1.911	0.072
Diabetes	66 (40.10%)	31 (25.20%)	2.085	0.018
Drug treatment			1.813	0.082
Clozapine	37 (22.98%)	24 (19.51%)		
Chlorpromazine	34 (21.12%)	28 (22.76%)		
Olanzapine	25 (15.53%)	21 (17.07%)		
Risperidone	58 (36.02%)	40 (32.52%)		
Quetiapine	4 (2.48%)	8 (2.44%)		
Other	3 (1.86%)	2 (1.63%)		
Course of disease (years)	26.51 ± 5.39	25.91 ± 6.22	6.124	0.055
FG (mg/dL)	96.12 ± 22.08	81.04 ± 17.12	9.088	0.004
Triglyceride (mg/dL)	198.09 ± 41.61	121.82 ± 40.72	27.046	0.013
HDL-C (mmol/L)	0.62 ± 0.24	1.04 ± 0.88	1.232	0.037
LDL-C (mmol/L)	2.68 ± 1.01	1.45 ± 0.96	1.055	0.016
Total cholesterol (mmol/L)	4.99 ± 1.76	4.13 ± 1.27	2.384	0.047
Apolipoprotein A1 (g/L)	0.96 ± 0.31	0.92 ± 0.19	1.131	0.112
Apolipoprotein B (g/L)	0.88 ± 0.27	0.61 ± 0.24	1.659	0.042
C-reactive protein (mg/L)	3.52 ± 2.09	3.24 ± 2.11	1.883	0.057
Uric acid (μmol/L)	4.31 ± 1.75	4.21 ± 1.62	2.005	0.108
Creatinine (μmol/L)	73.67 ± 13.14	74.18 ± 12.11	8.429	0.156
Urea nitrogen (mmol/L)	4.33 ± 1.28	4.22 ± 1.13	2.221	0.084
Total protein (g/L)	78.01 ± 24.43	76.58 ± 21.29	9.014	0.055
Albumin (g/L)	41.16 ± 9.26	44.92 ± 10.14	10.286	0.102
Globulin (g/L)	26.25 ± 11.07	26.15 ± 13.29	7.121	0.083
PANSS score	74.18 ± 12.09	84.61 ± 11.79	12.72	0.001

*BMI* body mass index, *FG* fasting glucose, *HDL-C* high-density lipoprotein cholesterol, *LDL-C* low-density lipoprotein cholesterol

### Pearson correlation analysis

As shown in Table 2, Pearson correlation analysis indicated that gender (*r* = 0.619), waist circumference (*r* = 0.644), BMI (*r* = 0.891), diabetes (*r* = 0.698), FG (*r* = 0.582), triglyceride (*r* = 0.618), HDL-C (*r* = −0.644), LDL-C (*r* = 0.583), apolipoprotein B (*r* = 0.595), and PANSS score (*r* = 0.813) were all correlated with the obesity in patients with schizophrenia (all *p* < 0.05).

**Table 2:** Pearson correlation analysis of obesity and related characteristics

Variables	*r*	*p*
Age (years)	0.113	0.058
Gender	0.619	0.021
Waist circumference (cm)	0.644	0.014
BMI (kg/m^2^)	0.891	0.002
Alcohol drinking	0.215	0.084
Smoking	0.174	0.121
Hypertension	0.155	0.074
Diabetes	0.698	0.039
Drug treatment	0.178	0.117
Course of disease (years)	0.106	0.092
FG (mg/dL)	0.582	0.045
Triglyceride (mg/dL)	0.618	0.012
HDL-C (mmol/L)	− 0.644	0.014
LDL-C (mmol/L)	0.583	0.027
Total cholesterol (mmol/L)	0.214	0.052
Apolipoprotein A1 (g/L)	0.106	0.088
Apolipoprotein B (g/L)	0.595	0.041
C-reactive protein (mg/L)	0.278	0.102
Uric acid (μmol/L)	0.124	0.058
Creatinine (μmol/L)	0.108	0.099
Urea nitrogen (mmol/L)	0.195	0.106
Total protein (g/L)	0.144	0.058
Albumin (g/L)	0.121	0.072
Globulin (g/L)	0.116	0.086
PANSS score	0.813	0.014

*BMI* body mass index, *FG* fasting glucose, *HDL-C* high-density lipoprotein cholesterol, *LDL-C* low-density lipoprotein cholesterol

### Logistic regression

The variable assignments of multivariate logistic regression are indicated in Table 3. As presented in Table 4, logistic regression analysis indicated that female (OR 2.129, 95% CI 1.615–3.022), waist circumference ≥ 90 cm (OR 3.814, 95% CI 2.778–4.312), diabetes (OR 2.856, 95% CI 1.905–3.448), FG ≥ 88 mg/dL (OR 1.551, 95% CI 1.284–2.183), triglyceride ≥ 160 mg/dl(OR 1.804, 95% CI 1.236–2.845), HDL-C ≤ 0.8 mmol/L(OR 2.032, 95% CI 1.614–3.079), LDL-C ≥ 2.0 mmol/L(OR 1.926, 95% CI 1.442–2.041) and apolipoprotein B ≥ 0.70 g/L (OR 2.119, 95% CI 1.658–2.873) were the independent risk factors of obesity in patients with schizophrenia (all *p* < 0.05).

**Table 3:** The variable assignments of multivariate logistic regression

Factors	Variables	Assignment
Obesity	*Y*	Yes = 1, no = 2
Gender	*X*_1_	Female = 1, male = 2
Waist circumference (cm)	*X*_*2*_	≥ 90 = 1, < 90 = 2
Diabetes	*X*_*3*_	Yes = 1, no = 2
FG (mg/dl)	*X*_*4*_	≥ 88 = 1, < 88 = 2
Triglyceride (mg/dl)	*X*_*5*_	≥ 160 = 1, < 160 = 2
HDL-C (mmol/L)	*X*_*6*_	≤ 0.8 = 1, > 0.8 = 2
LDL-C (mmol/L)	*X*_*7*_	≥ 2.0 = 1, < 2.0 = 2
Apolipoprotein B (g/L)	*X*_*8*_	≥ 0.70 = 1, < 0.70 = 2

FG, fasting glucose; HDL-C, high-density lipoprotein cholesterol; LDL-C, low-density lipoprotein cholesterol

**Table 4:** Logistic regression analysis on the risk factors of obesity

Variables	*β*	Wald	OR	95%CI	*p*
Female	0.208	0.119	2.129	1.615 –3.022	0.016
Waist circumference ≥ 90 cm	0.115	0.141	3.814	2.778 – 4.312	0.031
Diabetes	0.102	0.111	2.856	1.905 – 3.448	0.023
FG ≥ 88 mg/dl	0.314	0.217	1.551	1.284 –2.183	0.012
Triglyceride ≥ 160 mg/dl	0.187	0.129	1.804	1.236 – 2.845	0.047
HDL-C ≤ 0.8 mmol/L	0.104	0.164	2.032	1.614 – 3.079	0.011
LDL-C ≥ 2.0 mmol/L	0.181	0.122	1.926	1.442 – 2.041	0.024
Apolipoprotein B ≥ 0.70 g/L	0.155	0.116	2.119	1.658 – 2.873	0.031

*FG* fasting glucose, *HDL-C* high-density lipoprotein cholesterol, *LDL-C* low-density lipoprotein cholesterol

## Discussion

Patients with schizophrenia are prone to symptoms such as abnormal blood lipids, blood sugar levels, and obesity [18]. Costa-Dookhan and Kalinowska et al. [15, 19] have found that the obesity rate of schizophrenia patients is as high as 77.2%, of which 71.6% is male and 85.6% is female, which is 4 to 5 times that of the general population. Although different countries may have different definitions and measurement methods for obesity, they have all obtained consistent results that both outpatients and inpatients with schizophrenia generally have a higher incidence of obesity [20, 21]. Therefore, the obesity problem of schizophrenia patients has aroused the attention of mental health workers. We aimed at the obesity rate and related factors of deficit schizophrenia in the community. The results of this study have showed that the incidence of obesity in patients with schizophrenia is 56.70%. We can hypothesize that female, waist circumference ≥ 90 cm, diabetes, FG ≥ 88 mg/dL, triglyceride ≥ 160 mg/dL, HDL-C ≤ 0.8 mmol/L, LDL-C ≥ 2.0 mmol/L and apolipoprotein *B* ≥ 0.70 g/L may be the risk factors of obesity in patients with schizophrenia.

Hjorth et al. [22–24] have found that the obesity rate of inpatients with schizophrenia treated with clozapine is 36.11%. Mazereel et al. [25, 26] have reported that the obesity rate of inpatients with schizophrenia in Beijing is 20.9%. The above research results show that the obesity rate of patients with schizophrenia is higher than that of the normal population. In addition, Holt et al. [27–29] have shown that the worse the negative symptoms, the worse the patient's initiative, the less exercise, and the higher the body mass. In this study, the obesity rate of patients is higher than that of other studies, and the reason may be related to the study population in this study are patients with deficit schizophrenia with prominent negative symptoms.

The obesity rate of female patients is higher than that of male patients, and female, as an independent risk factor for obesity, increase the risk of obesity, which is consistent with the Oxenkrug’s results [30]. Most female patients in this study were in menopause or premenopause. Sarker et al. [31, 32] have shown that estrogen deficiency in women during menopause can lead to insulin resistance, abdominal obesity and related diseases. Besides, most antipsychotics will reduce female estrogen levels, and the decline in estrogen levels will lead to an increase in body weight, especially abdominal fat [33, 34]. Holt et al. [35, 36] have shown that the risk of obesity in postmenopausal women is 1.8 times that of premenopausal women. Therefore, the issue of obesity in menopausal women needs to be paid attention to.

Fasting blood glucose and fasting insulin levels in schizophrenia patients are significantly increased, and high insulin levels are high risk factors for obesity. However, insulin is a protein hormone secreted by islet cells stimulated by endogenous or exogenous substances such as glucose, lactose, ribose, arginine, and glucagon [37, 38]. Insulin can promote the synthesis and storage of fat, reduce free fatty acids in the blood, and inhibit the decomposition and oxidation of fat [39, 40]. Insulin deficiency can cause fat metabolism disorders, decreased fat storage, enhanced decomposition, and increased blood lipids [41]. However, Mizuki et al. [42, 43] have found that patients with schizophrenia often suffer from insulin resistance. It is speculated that due to insulin resistance, the uptake and utilization of glucose are decreased, and excess glycogen is converted into fat, which in turn leads to weight gain.

It shows that fasting blood glucose and triglyceride levels are risk factors for obesity. Ward et al. [44] have found that the incidence of metabolic syndrome in schizophrenia is higher than that of the normal population, which may be related to the use of atypical antipsychotics. Chen et al. [45–47] have found that typical antipsychotics and atypical antipsychotics can also affect the glucose and lipid metabolism of patients with schizophrenia. It has been reported that among the many second-generation antipsychotic drugs, olanzapine and clozapine have the most obvious effects on glucose and lipid metabolism [48, 49]. The effects and safety of antipsychotic drugs on obesity need further investigations in the future.

There are certain limitations in this study that deserve further consideration. Firstly, this study is a cross-sectional study. The enrolled patients were from a single city, and the representativeness of the sample was small. Studies with larger scale and more sample size are needed to confirm the results of this study. Secondly, the time elapsed since the first diagnosis of schizophrenia should be taken into consideration: a diagnosis of schizophrenia for 3 years or 25 years can lead to significant differences in obesity, we did not find the group difference limited by small sample size. Thirdly, we did not consider the factors such as family environment, eating habits, food intake, etc., of the enrolled patients, which may have a certain degree of influence on the obesity, which needs further evaluated in future studies.

## Conclusions

In summary, we can hypothesize that the incidence of obesity in patients with schizophrenia is 56.70%. Female, waist circumference ≥ 90 cm, diabetes, FG ≥ 88 mg/dL, triglyceride ≥ 160 mg/dL, HDL-C ≤ 0.8 mmol/L, LDL-C ≥ 2.0 mmol/L and apolipoprotein *B* ≥ 0.70 g/L may be the risk factors of obesity. Due to the limitations of various factors and conditions, how to adjust and follow up the weight monitoring and management of patients in the community after they are discharged from the hospital and returned to the family is still the major problems currently facing. Exploring a scientific and reasonable comprehensive weight management model for patients with schizophrenia from the hospital to the family and the community is an important topic worthy of in-depth study in the future.

## Data Availability

All data generated or analyzed during this study are included in this published article.

## Abbreviations

BMI: Body mass index
HDL-C: High-density lipoprotein cholesterol
LDL-C: Low-density lipoprotein cholesterol
PANSS: Positive and Negative Symptom Scale
